# Space Use and Movement of a Neotropical Top Predator: The Endangered Jaguar

**DOI:** 10.1371/journal.pone.0168176

**Published:** 2016-12-28

**Authors:** Ronaldo G. Morato, Jared A. Stabach, Chris H. Fleming, Justin M. Calabrese, Rogério C. De Paula, Kátia M. P. M. Ferraz, Daniel L. Z. Kantek, Selma S. Miyazaki, Thadeu D. C. Pereira, Gediendson R. Araujo, Agustin Paviolo, Carlos De Angelo, Mario S. Di Bitetti, Paula Cruz, Fernando Lima, Laury Cullen, Denis A. Sana, Emiliano E. Ramalho, Marina M. Carvalho, Fábio H. S. Soares, Barbara Zimbres, Marina X. Silva, Marcela D. F. Moraes, Alexandre Vogliotti, Joares A. May, Mario Haberfeld, Lilian Rampim, Leonardo Sartorello, Milton C. Ribeiro, Peter Leimgruber

**Affiliations:** 1 Centro Nacional de Pesquisa e Conservação de Mamíferos Carnívoros, Instituto Chico Mendes de Conservação da Biodiversidade, Atibaia, São Paulo, Brazil; 2 Conservation Ecology Center, Smithsonian Conservation Biology Institute, National Zoological Park, Front Royal, Virginia, United States of America; 3 Instituto Pró-Carnívoros, Atibaia, São Paulo, Brazil; 4 Escola Superior de Agricultura “Luiz de Queiroz”, Universidade de São Paulo, Piracicaba, Brazil; 5 Estação Ecológica Taiamã, Instituto Chico Mendes de Conservação da Biodiversidade, Cáceres, Mato Grosso, Brazil; 6 Departamento de Medicina Veterinária, Universidade Federal de Viçosa, Viçosa, Minas Gerais, Brazil; 7 Instituto de Biología Subtropical, Universidad Nacional de Misiones and CONICET, Puerto Iguazú, Argentina; 8 IPÊ – Instituto de Pesquisas Ecológicas, Nazaré Paulista, São Paulo, Brazil; 9 Laboratório de Ecologia Espacial e Conservação, Instituto de Biociências, Univesidade Estadual de São Paulo, Rio Claro, São Paulo, Brazil; 10 Programa de Pós-graduação em Ecologia, Universidade Federal do Rio Grande do Sul, Porto Alegre, Rio Grande do Sul, Brazil; 11 Instituto de Desenvolvimento Sustentável Mamirauá, Tefé, Amazonas, Brazil; 12 Instituto de Defesa e Preservação dos Felídeos Brasileiros, Corumbá de Goiás, Goiás, Brazil; 13 Programa de Pós Graduação em Zoologia, Instituto de Ciências Biológicas, Universidade de Brasília, Federal District, Brazil; 14 Projeto Carnívoros do Iguaçu, Parque Nacional do Iguaçu, Instituto Chico Mendes de Conservação da Biodiversidade, Foz do Iguaçu, Paraná, Brazil; 15 Universidade Federal da Integração Latino-Americana, Foz do Iguaçu, Paraná, Brazil; 16 Projeto Onçafari Miranda, Mato Grosso do Sul, Brazil; Leibniz-Institute of Freshwater Ecology and Inland Fisheries, GERMANY

## Abstract

Accurately estimating home range and understanding movement behavior can provide important information on ecological processes. Advances in data collection and analysis have improved our ability to estimate home range and movement parameters, both of which have the potential to impact species conservation. Fitting continuous-time movement model to data and incorporating the autocorrelated kernel density estimator (AKDE), we investigated range residency of forty-four jaguars fit with GPS collars across five biomes in Brazil and Argentina. We assessed home range and movement parameters of range resident animals and compared AKDE estimates with kernel density estimates (KDE). We accounted for differential space use and movement among individuals, sex, region, and habitat quality. Thirty-three (80%) of collared jaguars were range resident. Home range estimates using AKDE were 1.02 to 4.80 times larger than KDE estimates that did not consider autocorrelation. Males exhibited larger home ranges, more directional movement paths, and a trend towards larger distances traveled per day. Jaguars with the largest home ranges occupied the Atlantic Forest, a biome with high levels of deforestation and high human population density. Our results fill a gap in the knowledge of the species’ ecology with an aim towards better conservation of this endangered/critically endangered carnivore—the top predator in the Neotropics.

## Introduction

### Top predator as a model

In terrestrial environments, predators tend to restrict their movements within defined areas to meet daily requirements. These animals are often considered range residents [1]. Top predators, such as large-sized cats, are known to require large areas [2]. Space use is likely to increase as habitat quality decreases, making this group particularly vulnerable to habitat loss and fragmentation [3–5]. In addition, movement parameters such as the total distance traveled per day and the tortuosity of the movement path can increase in response to habitat fragmentation [6]. Understanding animal movement and space use across dynamic landscapes is critical for the establishment of effective conservation strategies [7], including the creation/maintenance of ecological corridors designed to guarantee the movement of focal species, improving the connectivity of habitat patches within fragmented landscapes [8], and identifying priority areas for conservation [9]. Accurately estimating home ranges and understanding animal movement behavior provide information on ecological processes that can impact species conservation [10,11].

The jaguar (*Panthera onca*) is widely distributed across a broad range of habitats in the Americas [12]. Loss of habitat is the greatest threat for the species’ long-term survival. The classification of the species as “near threatened” by the IUCN [13] and endangered or critically endangered in Brazil and Argentina [14,15] reflects that over 50% of the species’ natural habitat has been lost and converted to anthropogenic land-uses in the past century [12].

Jaguars are known to be range resident with young dispersing from their natal area after becoming independent [16]. Several studies have estimated jaguar home range across the species’ distributional range [17–20]. Nevertheless, no study has accounted for the inherent autocorrelation structure of the movement data when calculating jaguar home range estimates. Resulting home ranges are likely underestimated [11]. Moreover, former studies lacked an empirical way to characterize range residency. Thus, published results could include estimates of “home range” for individuals that are not actually range resident but may be dispersing or transient. It remains unclear if study-specific differences in jaguar home range estimates are ecologically-based or the result of methodological artifacts [18]. Surprisingly few studies have investigated jaguar movement to date [16,21–23], due in part to the difficulty in locating and fitting individuals with monitoring devices. Differential movement strategies have been reported between sexes, with males moving greater distances and females being more restricted to home range center points [21,24]. However, more detailed analyses, utilizing quickly evolving and advanced analytical tools from movement ecology, are urgently needed on the movements of jaguar across differing habitats, particularly across differing degrees of habitat disturbance and fragmentation.

### New perspectives on movement data analysis

The minimum convex polygon (MCP) and kernel density estimators (KDE) are the most commonly used tools to estimate animal home ranges [11]. Both tools have limitations that are often not acknowledged. MCPs, for example, lack any underlying probabilistic model while KDEs are derived under the assumption of independent and identically distributed data (IID), a process that assumes uncorrelated positions and velocities and which cannot be recognized as a movement model [11,25]. Incorporating autocorrelated data in conventional KDE frameworks has the potential to underestimate the size of animal home ranges, especially as the temporal frequency of positions collected increases [11]—a situation common with modern GPS tracking devices.

New approaches such as fitting continuous-time stochastic movement models to animal tracking data can account for inherent serial autocorrelation [26]. Movement analyses using this method have a number of desirable properties, including the ability to handle irregular sampling schedules (including gaps in the data) and complex autocorrelation structures [25,27–29]. This approach includes variogram analysis [27] and non-Markovian maximum likelihood estimation [28]. The former facilitates identifying important features in the data (e.g., range residency), while the latter allows models incorporating these features to be rigorously fit to the data. Once an appropriate continuous-time model has been selected and fit, Autocorrelated Kernel Density Estimation (AKDE) then conditions on the fitted model to allow accurate home range estimation even when data are strongly autocorrelated [11]. Calabrese *et al*. (2016) [25] give a detailed account of using the continuous-time movement modeling (*ctmm*) R package to perform this sequence of analyses.

### Jaguar motion in focus

We used the AKDE method to estimate home range, to evaluate path tortuosity, and to quantify average distance traveled by jaguar across different regions of Brazil and Argentina. Our first step involved characterizing the underlying movement behavior. We expected adult jaguars to be range resident. Second, we expected larger home range estimates than previously reported for the species, due to appropriately accounting for the autocorrelation structure of the data [17,20,30–33]. We calculated estimates of home range using both KDE and AKDE methods, demonstrating ramifications. We also expected differential space use and movement between individuals and sexes [18,23]. We predicted that jaguar inhabiting regions with poor habitat quality (e.g., areas with high human presence and high levels of habitat loss) to have larger home ranges. Animals throughout these regions should exhibit more directional and persistent movement, with greater average distance traveled per day [6].

## Materials and Methods

### Study area and data collection

**Fig 1:**
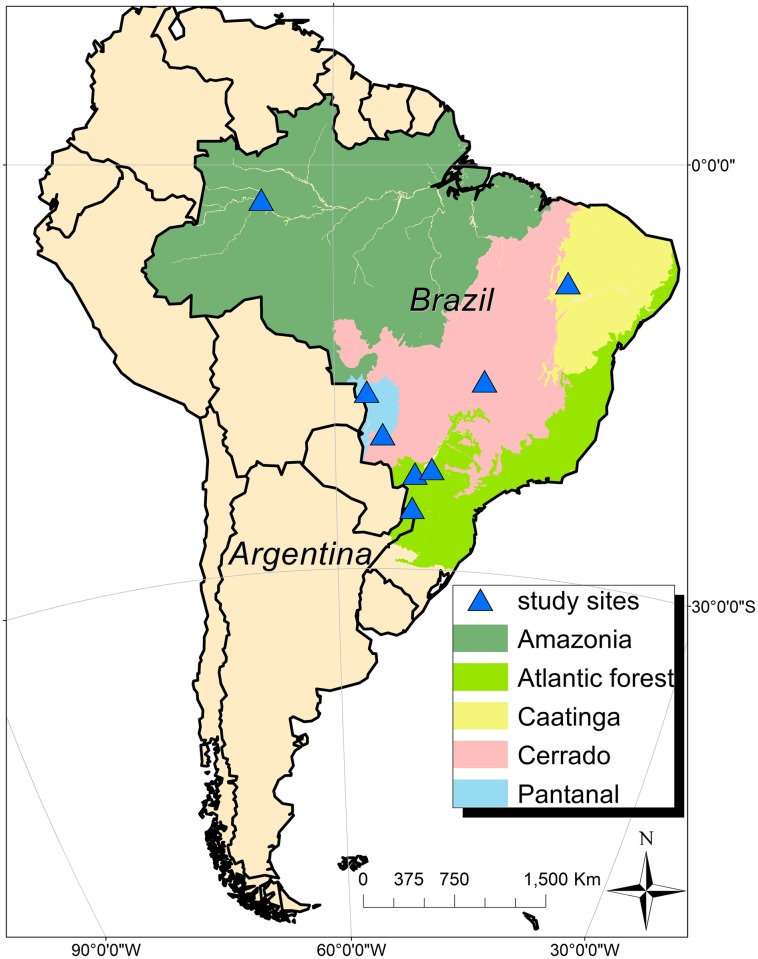
Map of study areas in Brazil and on the border of Brazil and Argentina. Source: mma.gov.br and wwf.org

We used GPS tracking to monitor 44 Jaguars from 1998 to 2016 and across different habitats representing five biomes in Brazil and Argentina. Our dataset represents the largest collection of jaguar movement data analyzed to date. Biomes included the Amazon (4 males: 4 females), Atlantic Forest (6:6), Caatinga (2:0), Cerrado (1:0), and Pantanal (9:12) (Fig 1 and Table 1).

**Table 1:** Extent and conservation status of remaining habitat in Brazil’s major biomes and a portion of Atlantic Forest in Argentina. Jaguars are considered vulnerable in the Amazon and Pantanal, endangered in the Cerrado and critically endangered in the Atlantic Forest and Caatinga [14,15].

	Amazon	Atlantic Forest	Caatinga	Cerrado	Pantanal
Biome area (km^2^)	4,196,943	1,110,182	844,453	2,036,448	150,355
Percentage of Brazil (%)	49.3	13.0	9.9	23.9	1.8
Biome remaining (%)	82.3	12.0	52.5	51.6	84.7
Protected Areas (%)	49.1	9.6	7.7	12.3	4.6
Mean habitant density per Km^2^	3	77	13	5	3
Mean livestock density per Km^2^	0.15	21.8	8.0	48	32.2
Jaguar Density (individuals per 100km^2^)	10.0	0.45–2.2	2.67	2.0	10.3

Source: http://siscom.ibama.gov.br/monitorabiomas/, [18,34–37].

The GPS collection schedules and time periods each animal was monitored ranged from one position every half hour to one position every 24 hours. Estimated ages of jaguars ranged from 18 months to 10 years, with the majority of jaguar (*n* = 41) being adults (> 3 years old). Two individuals (Esperança and Xango) were monitored for two different periods. Monitoring periods ranged from 11 to 1,749 days (mean = 183 days), while the number of recorded locations ranged from 53 to 10,989 (mean = 2,264). The total dataset consisted of 80,553 locations. Further details on fix schedules, the number of days each animal was monitored, and the devices used to monitor movement, are provided in S1 Table. All animals were captured following standard protocols approved by the Instituto Chico Mendes de Conservação da Biodiversidade—Ministério do Meio Ambiente—Brazil (ICMBio-SISBIO license numbers: 30896–3, 46031–4, 36740–1, 44677–1,14202–4, 38006–1, 30053–1, 37867–1), the National Park Administration (NPA license 03/09), and Misiones Province Government (ME license 119/2012) from Argentina.

Collar fitting involved using trained dogs [38], box traps [30] and/or foot snares [39] to facilitate animal capture. All individuals were anaesthetized with a combination of tiletamine and zolazepam (10 mg kg^-1^), administered via an aluminum dart fired from an air-powered rifle [38]. We examined each immobilized animal for general body condition, determined its sex and age, collected its weight, and fit each jaguar with a global positioning system collar. Animals were released at the site of capture. All procedures followed guidelines approved by the American Society of Mammologists [40].

### Home range and movement

We calculated variograms, fit movement models, and estimated home ranges using the *ctmm* package [25,41] in the R environment for statistical computing [42]. For each animal, we plotted the estimated semi-variance (function *variogram*) as a function of time lag to visually inspect the autocorrelation structure of the location data [27]. At zero to short time lags, a linear increase in the semi-variance corresponds to uncorrelated velocity, suggesting movement models such as Brownian motion (BM) or Ornstein-Uhlenbeck (OU). Upward curvature at these time lags indicates velocity autocorrelation and suggests movement models such as Integrated OU (IOU) or OU with foraging (OUF) [25,27]. Space use was investigated by inspecting the behavior across longer time lags. Range residents are expected to reach an asymptote on a timescale that roughly corresponds to the home-range crossing time [25,27]. Individuals whose plotted semi-variance did not approach an asymptote, however, were not assumed to be range resident [25]. These animals were either not monitored for a long enough time period or did not exhibit behaviors that meet the definition of a range resident and were removed from further analysis.

Models were fit via maximum likelihood (function *ctmm*.*fit*) [28] and ranked based on AICc [43] (function *ctmm*.*select*) [25]. We estimated home range conditional on the fitted, selected model for each individual using the *akde* function. OU models are described by two parameters [i.e., home range crossing time (days) and variance (km^2^)], while OUF models are described by three parameters [i.e., home range crossing time (days), velocity autocorrelation timescale (h), and variance (km^2^)]. These procedures resulted in estimates of the home range, home range crossing time, velocity autocorrelation timescale and average distance traveled for each individual if the selected model was OUF, or home range and home range crossing time if the best model was OU. To show that KDE underestimates home range, we calculated conventional KDEs (95%) for each animal, also fit in *ctmm* by passing a fitted Independent Identically Distributed (IID) model (i.e., a model that, by definition, ignores autocorrelation in the data) to the *akde* function [25].

### Statistical analyses

To test our predictions that animal space use and movement (i.e., home range, home range crossing time, velocity timescale and average distance traveled) varied with gender and biome (i.e., Atlantic rainforest vs. Pantanal vs. Amazon), we compared results using hierarchical Bayesian fixed-effect one-way ANOVAs [44,45]. We tested for normality using Shapiro-Wilk tests [46] and by visually inspecting Q-Q plots. Data were log transformed if dataset distributions did not meet model assumptions. We estimated marginal posterior distributions of parameters using Markov chain Monte Carlo (MCMC) methods. We fit a MCMC algorithm with 100,000 iterations and a 20% burn-in period (i.e., 20,000 iterations). We assessed convergence by visually inspecting trace plots to ensure a reasonable exploration of the parameter space and by confirming that the potential scale reduction factor was <1.02 for each variable [47]. We calculated the probability (*P*) that the mean of one group was greater than the other by sampling from each of the resulting posterior distributions (10,000 iterations) [48]. We implemented all analyses in program R using the *rjags* package [49], JAGS version 4.2.0.

We used human population (*LandScan* [50]) as a corollary of habitat quality assuming that as human population density increases, habitat quality decreases [51,52]. To assess the effect of human population (square root transformed) on home range estimates, we fit linear regression models in a Bayesian framework. Models were implemented in JAGS 4.2.0 [52] in the R programming language following methods previously described [44,53]. We assessed model fit by calculating the Bayesian *p*-value—the proportion of times when the replicated “ideal” dataset is greater than the actual dataset [44,48]. Values close to 0.50 indicate a good model fit (i.e., no difference between the two datasets). Model variability was displayed by randomly sampling (10,000 times) from the posterior distributions of the alpha and beta parameters.

## Results

### Range residency of jaguars

**Fig 2:**
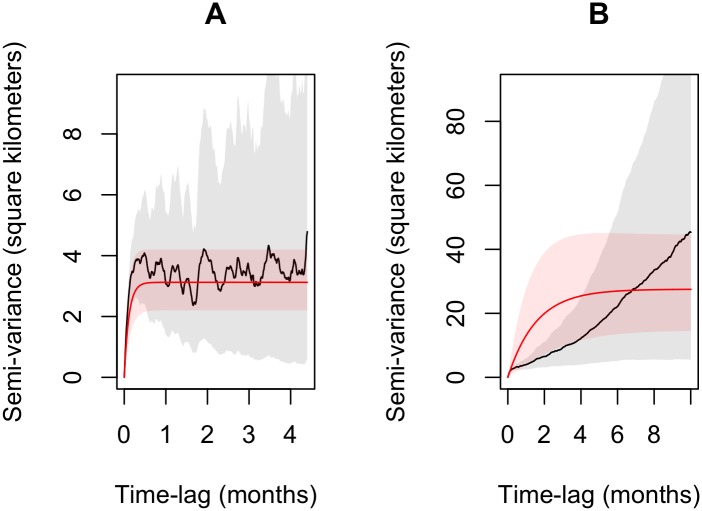
(A) Variogram of a resident jaguar. Notice that the animal’s semi-variance reaches an asymptote within a few days, roughly representing the time to cross its home range. The red line represents the fitted model and the red shading represents the 95% CI. (B) A non-resident jaguar. Note the lack of a clear asymptote despite the fact that the animal was monitored for a long period (591 days). This lack of asymptote indicates that this animal is not range resident and thus a home range analysis for this individual is not appropriate. For both A and B, the fraction of the variogram displayed is 65% of the duration of each dataset.

Thirty-three (33) individuals (15 males and 18 females) were determined to be range resident after variogram inspection. Eight individuals (5 male, 3 female) were assumed to be non-residents (Fig 2). Three individuals were monitored for too short of a period (< 27 days) to determine movement behavior. Comparative analyses across biomes excluded animals from the Caatinga and Cerrado since no animals were considered residents (Caatinga) or due to a lack of an adequate sample size (Cerrado).

### Individual home range and movement of jaguar

#### Comparison between AKDE and KDE

Home range and movement (home range crossing time, velocity timescale, and average distance traveled) estimates varied between individuals (Table 2). Home range estimates using AKDE were 1.0 to 4.8 times larger than estimates obtained using KDE. Other than a few exceptions, AKDE estimates were larger than estimates previously reported (Table 3).

**Table 2:** Movement parameters and home range sizes for GPS-collared jaguar across Brazil and Argentina biomes. Home ranges were estimated via 95% Kernel Density Estimates (KDE) and Autocorrelated Kernel Density Estimates (AKDE)^1^.

ID	Sex/age (years)	Number of fixes/days	Home range crossing time (day)	Velocity autocorrelation timescale (h)	Average distance traveled (km/day)	95% KDE (km^2^)^2^	AKDE (km^2^) (95% CI)
*Amazon*							
Baden	M/9	1,024/507	6.8	2.6	4.4	169.5	207.0 (168.8–249.3)
Caculao	M/7	516/190	5.9	3.3	4.6	180.3	253.7 (187.7–329.6)
Confuso	M/9	61/251	3.4	1.9	4.3	67.6	75.9 (39.3–124.5)
Coto	F/7	501/154	9.7	2.5	2.3	53.0	85.5 (52.9–125.9)
Mamad*	M/7	295/383	20.4	NA	NA	174.3	309.7 (167.2–495.3)
Mamae	F/11	784/333	4.8	0.9	4.2	43.7	49.4 (41.3–58.2)
Mudinha	F/5	3,700/429	7.7	1.0	3.9	53.6	70.2 (58.1–83.4)
*Atlantic Forest*							
Cassio	M/6	159/159	1.5	NA	NA	108.5	110.9 (92.1–131.4)
Denis	M/5	797/370	4.5	0.9	15.4	414.9	502.1 (435.9–572.9)
Femea*	F/5	211/139	4.3	NA	NA	85.6	113.1 (85.2–145.0)
Gigi	F/7	35/1,749	2.6	NA	NA	233.5	246.2 (164.4–344.3)
Livia*	F/7	183/209	18.5	NA	NA	230.4	718.6 (312.9–1290.1)
Taia*	F/4	326/1,141	7.3	NA	NA	183.2	250.7 (187.4–323.1)
Guacurari	M/7	7,668/220	6.2	0.5	15.3	421.4	560.8 (431.7–706.6)
Naipi*	F/2	53/119	2.5	NA	NA	137.6	143.8 (98.8–197.0)
Yasirandi	F/6	322/224	1.6	2.2	7.0	134.5	135.6 (117.0–155.5)
Zezao*	M/8	156/171	2.1	NA	NA	591.4	677.4 (550.7–817.1)
*Cerrado*							
Xango 1	M/?	1,633/153	6.9	0.8	18.3	722.5	1,268.6 (831.9–1,795.8)
Xango 2	M/?	799/179	4.5	1.9	14.3	807.4	1,163.2 (904.8–1,453.6)
*Pantanal*							
Anderson	M/7	5,040/260	3.3	0.3	8.7	25.0	37.2 (32.1–47.2)
Caiman	M/5	2,303/135	4.5	0.4	8.9	70.8	144.0 (78.9–144.0)
Dale	M/7	4,705/252	9.3	0.3	6.7	58.4	91.9 (66.3–121.7)
Fera	**F/3**	**4,952/255**	**4.7**	**0.4**	**5.7**	**25.2**	**34.8 (30.2–39.7)**
Milagre	M/6	3,339/191	12.8	0.4	7.2	54.7	174.3 (105.0–261.0)
Selema	F/6	2,817/126	4.2	0.4	5.8	23.7	37.8 (28.1–48.8)
Wendy*	F/5	1,287/192	8.1	NA	NA	27.4	52.1 (36.0–71.2)
Brutus	M/5	1,256/76	3.6	0.5	15.6	193.2	277.7 (189.3–382.8)
Chuva	F/10	741/73	0.9	0.3	13.9	31.5	35.9 (29.1–43.4)
Esperanca 1	F/7	842/53	1.1	0.2	15.4	31.1	39.7 (31.8–48.3)
Esperanca 2	F/10	2,232/126	1.8	0.2	12.5	31.2	36.9 (31.5–42.7)
Nati	M/10	758/52	2.5	0.4	15.8	98.1	175.5 (113.6–259.7)
Nusa	F/10	2,201/127	2.5	0.4	8.9	46.5	58.0 (47.5–69.7)
Teorema	F/7	4,643/275	2.3	0.3	11.4	50.4	61.0 (54.9–67.4)
Troncha	F/10	1,324/87	2.8	0.3	14.3	111.2	138.6 (102.2–180.3)
Vida	F/5	398/33	0.6	0.3	16.4	15.3	24.7 (19.2–30.9)

^1^We used ctmm for AKDE home range estimation, following procedures by Fleming et al. (2015) [11] and Calabrese *et al*. (2016) [25]. For most animals we were able to fit an Ornstein-Uhlenbeck Foraging (OUF) process model (Fleming et al 2014a, b) [27,28] to estimate home range area. Home ranges for animals marked with * were based on an Ornstein-Uhlenbeck (OU) process model.

^2^Confidence intervals can be estimated for KDE using the *ctmm* package [25]. These data, however, were small and not included.

**Table 3:** Jaguar home range estimates from the Amazon, Atlantic Forest, Cerrado, and Pantanal using the autocorrelation kernel density estimator (AKDE), minimum convex polygon (MCP), or kernel density estimator (KDE). For AKDE, MCP, and KDE we display the mean, minimum, and maximum home range values. For AKDE, we also display 95% confidence intervals.

Biome	Method	Home range (km^2^)	Mean home range (km^2^)		Reference
			Female	Male	
Amazon	AKDE	49.4–309.7	68.4 (23.3–113.4) (n = 3)	211.6 (52.9–370.2) (n = 4)	This study
Atlantic Forest	AKDE	110.9–718.6	268.0 (223.1–702.4) (n = 5)	462.8 (71.9–853.7) (n = 4)	This study
Atlantic Forest	MCP 100%	8.8–138	39.4 (n = 2)	88.7 (n = 4)	[30]
Atlantic Forest	MCP 100%	43.8–177.7	87.3 (n = 5)	102 (n = 2)	[31]
Atlantic Forest	KDE 85%	87–173	130 (n = 2)	147 (n = 1)	[38]
Cerrado	AKDE	NA	NA	1,268.6 (831.9–1,795.8) (n = 1)	This study
Cerrado	MCP 80%	228–265	228 (n = 1)	265.2 (n = 2)	[33]
Pantanal	AKDE	24.7–277.7	52.0 (28.7–75.2) (n = 10)	144.3 (56.3–232.2) (n = 6)	This study
Pantanal	MCP 100%	25–90	32.3 (n = 3)	90 (n = 1)	[16]
Pantanal	MCP 100%	97.1–168.4	139.6 (n = 4)	152.4 (n = 1)	[54]
Pantanal	Kernel 95%	NA	38.2 (n = 5)	67.4 (n = 3)	[19]

Adapted from Astete et al. (2007) [18].

#### Sex differences on home range and movement parameters of jaguars

**Fig 3:**
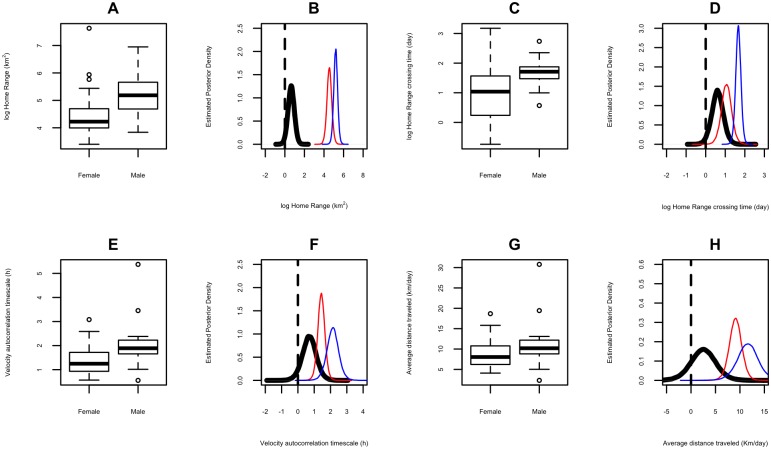
Boxplot and Posterior Density Estimates for male and female home range (log km^2^) [A and B], home range crossing time (log days) [C and D], velocity autocorrelation timescale (h) [E and F], and average distance traveled (Km/day) [G and H]. Black line represents the difference between the posterior distribution of males and females, red represents the posterior distribution of females and blue represents the posterior distribution of males.

Movement and home range size varied widely between male and female jaguar. The probability that the home range size of males (range: 37.2 to 1,268.6 km^2^) was greater than females (range: 24.7 to 718.6 km^2^) was 0.97 (Fig 3A and 3B). Males, almost exclusively, took longer to cross their home ranges (*P* = 0.99; Fig 3C and 3D). Male movement paths, represented by velocity autocorrelation timescale, were proportionally more directional (*P* = 0.94; Fig 3E and 3F), with a greater distance traveled per day (*P* = 0.84; Fig 3G and 3H) when compared with female jaguar. All data are summarized in Table 2.

#### Differences in home range and movement across areas with differing degrees of habitat loss and human population density

**Fig 4:**
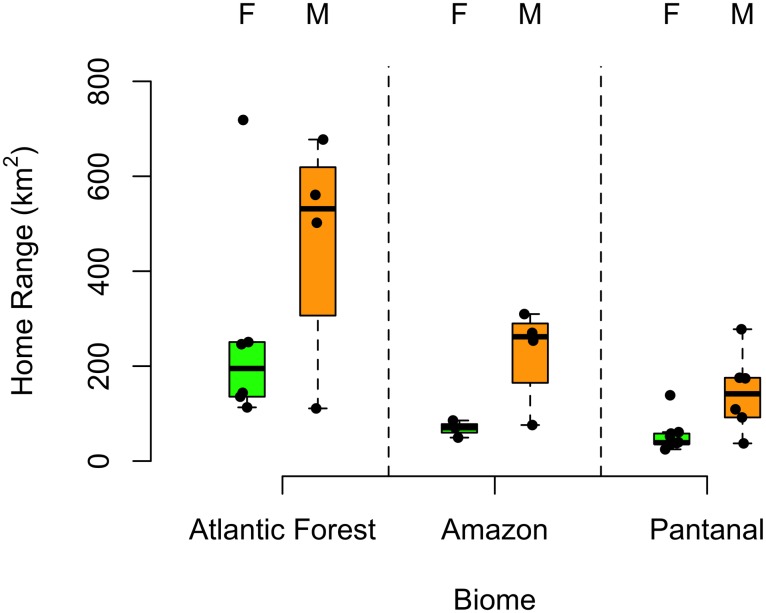
Boxplot of home range (km^2^) for males and female jaguar by biome.

We observed differences in home range size between biomes (Fig 4). The probability that the home ranges of Atlantic Forest male jaguar were greater than individuals from the Amazon or Pantanal was 0.87 and 0.98, respectively. Similarly, the probability that the home range of Atlantic Forest female jaguar were greater than individuals from the Amazon or Pantanal was 0.99 and 1.0, respectively.

Differential movements were observed across biomes. Female jaguar took 9.0 days (95% CI: 4.7–13.4), 7.4 days (95% CI: 1.3–9.4) and 2.3 days (95% CI: 0.1–6.4) to cross their home range in the Atlantic Forest, Amazon and Pantanal, respectively. The probability that the velocity autocorrelation timescale of female Amazon jaguar was greater than female Pantanal jaguar was 0.89. The inverse, however, was observed in relation to daily distance traveled (Pantanal > Amazon). The home range crossing time for male Amazon jaguar was greater than male jaguar from the Pantanal or Atlantic Forest, although male jaguar from Pantanal took more days to cross their home range than animals from the Atlantic Forest (Amazon > Pantanal > Atlantic Forest). A similar result was found for the velocity autocorrelation timescale. Average distance traveled was highest for jaguar inhabiting the Atlantic Forest (Atlantic Forest > Pantanal > Amazon) (Table 4).

**Table 4:** Probability that the home range and movement parameter mean of male and female jaguars was different among locations (row vs column).

	Amazon				Pantanal			
	Home Range (km^2^)	Home range crossing time (day)	Velocity autocorrelation timescale (h)	Average Distance traveled (km/day)	Home Range (km^2^)	Home range crossing time (day)	Velocity autocorrelation timescale (h)	Average Distance traveled (km/day)
*Male*								
**Amazon**					0.85	0.85	0.99	0.02
**Atlantic Forest**	0.87	0.04	0.02	0.99	0.98	0.18	0.98	0.90
*Female*								
**Amazon**					0.88	0.97	0.89	0.01
**Atlantic Forest**	0.99	0.66	NA	NA	1.0	0.98	NA	NA

NA- Not applicable, insufficient data.

**Fig 5:**
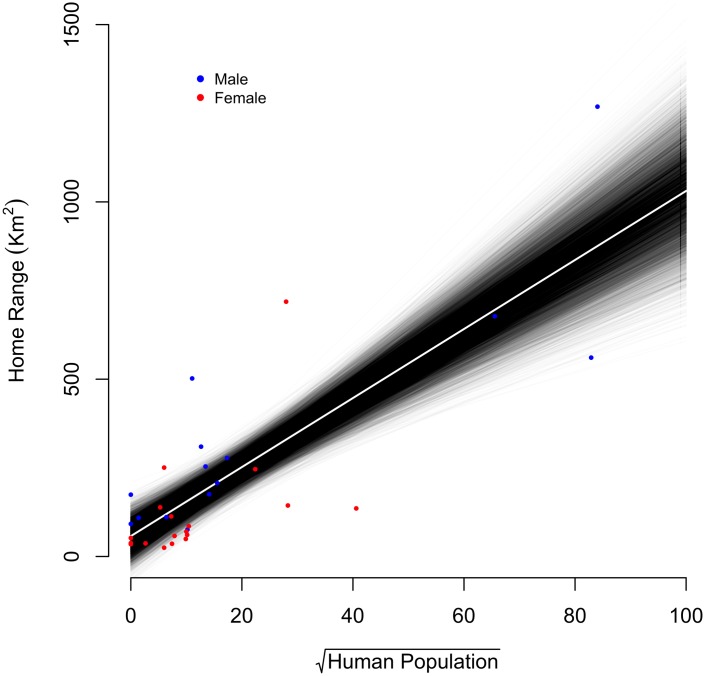
Jaguars’ home range estimates in relation to human population size (square root transformed) across four study areas in Brazil and Argentina. Regression line is the species estimate from a linear regression model formulated in a Bayesian framework (Bayesian p-value = 0.495). Error lines are 95% CI.

Jaguar home ranges also increased in size with increasing human population (Fig 5). Bayesian *p*-value (0.495) indicates an adequate fit of the regression model to the data. Males were most affected by human population size, represented by increases in space use. The response of female jaguar was more restricted, with few home ranges showing increases in relation to increases in human population. Largest home ranges for male and female jaguar were observed across the Atlantic Forest and Cerrado.

## Discussion

The Autocorrelated Kernel Density Estimator is a recent analytical development in movement ecology that removes the negative bias in home range estimation by incorporating the autocorrelation structure inherent in most movement datasets. This method yields better home range estimates, allowing movement models to be fit to data with different temporal structures (e.g., irregular sampling intervals, gaps, and short periods of data collection) [11]. Using this flexible approach, we revealed important ecological processes in jaguar, including heterogeneity in space use and movement owing to differences in individual, gender, region, and habitat quality.

Our results provide support for differential movement behavior and space use between individuals and sex. Additional differences were observed between regions with differing degrees of human disturbance (i.e., population density), revealing important aspects of jaguar ecology. Most large felids are broadly distributed and inhabit different habitat types [2]. Habitat loss and increased human disturbance have posed several threats to these species [2]. Our approach can help in providing better information on the movement ecology of these species, resulting in an important contribution to long-term conservation and management.

### Jaguar residency

Several jaguars we tracked never established a home range. For juvenile jaguars (< 3 years old), this may not be surprising [16]. But, we also discovered non-resident movement behavior in five adult jaguars (> 4 years old). Azevedo and Murray (2007) [32] considered an animal ‘resident’ when it was observed to stay in the same area for at least 2 years. Such arbitrary classification may not be accurate. In our study, we found a non-resident adult individual that had been monitored for 591 days (S1 Table). Consequently, time spent in an area is not adequate to estimate home range and cannot explain how and why an individual uses space [55]. In contrast, observing a clear asymptote in the variogram of an animal’s observed movement track provides objective evidence of range residency [25,28].

Recent research in movement ecology demonstrates that individuals of the same species may exhibit different movement strategies under different environmental conditions [56]. This seems to be well established for herbivores, such as wildebeest, that can be migratory, nomadic or range resident [57,58]. Similar behavioral and environmental plasticity have also been reported for carnivore species such as lion [59], polar bear [60], wolverine [61], and wolf [62]. In our study, we did not identify the underlying movement behavior of non-resident jaguars, observed in jaguar collared in the Pantanal and the Caatinga biome. Non-resident jaguar inhabiting the Pantanal exhibited a more directed linear movement path with “short stops”. Non-resident jaguar inhabiting the Caatinga remained stationary for long periods (2–3 months) before dispersing long distances (> 50 km) and returning to their original location (data not shown). These movements could be described as nomadic (as has been described for lions [59]) or potential migratory behavior. Further investigation is required.

### AKDE vs KDE

Our home range estimates using AKDE are larger than those reported in the past and when compared with KDE estimates calculated on the same data (see Tables 2 and 3). Although one might expect some variation in home range size when monitoring different individuals [17,19,20,54], differences observed between previous estimates are consistent and most likely represent the difference in how the autocorrelation structure of the data was incorporated. Both MCP and KDE methods ignore autocorrelation and have been proven to underestimate home range area when used on autocorrelated tracking data [11,27]. AKDE accounts for autocorrelation in the data and adjusts home range estimates accordingly (and with appropriate confidence intervals) [32]. An accurate estimate of the home range can result in vital insight into ecological processes [10] and provides a promising avenue for further investigation. Most importantly, our estimates highlight that management plans based on previous published results could severely underestimate the amount of area required to adequately protect the species.

### Males vs females

Our findings that male jaguar have larger home ranges than females is consistent with results from previous studies of jaguar space use [18,20,23]. A larger home range in terrestrial male carnivores has been suggested to be bounded by the distribution of females and the need to increase mating/reproductive opportunities [63]. Female home range size is known to be shaped by the distribution of food availability, which is particularly important for successful reproduction, including gestation and care of offspring [65]. Our findings reinforce these observations, with female jaguar movement paths being proportionally more tortuous (represented by smaller velocity autocorrelation timescale) with smaller average distances traveled per day when compared with males that are likely to take greater risks.

### Differences in home range and movement across areas with differing degrees of habitat loss and human population density

Although differences in space use and movement have been reported for species inhabiting different regions [56], this is the first study to report differential space use and movement of jaguar across areas with different degrees of habitat loss and human population density. Jaguars inhabiting the most disturbed biome, the Atlantic Forest—with only 12% of habitat remaining and with high human population density, resulted in large home range sizes and an increased average distance traveled. Similarly, one jaguar inhabiting the Cerrado, a biome that has lost 50% of its natural area [36], had the largest home range observed (1,268.6 km^2^).

We provide a broad overview of factors that can influence jaguar movement decisions. At finer scales, several human activities such as livestock production, poaching, and roads [52,64] influence jaguar space use and movement. Colchero et al. (2011) [22] observed that human population density and roads have strong effects on female jaguar movement decisions. Male jaguar, however, were less affected. In our study, jaguar space use increased in areas with higher human population presence and higher levels of habitat loss. While jaguar have been frequently reported to prey on livestock [65], it is unclear if high livestock densities affect jaguar movement decisions. Kanda (2015) [24], for example, observed that livestock did not influence jaguar movement decisions. Instead, social interactions were reported to be the main factor impacting jaguar movements. In our study, we were limited by the resolution of the livestock density dataset (10km^2^) and therefore, did not evaluate the response of jaguar to livestock. Incorporating a finer resolution dataset of livestock density (< 1km^2^) combined with resource selection or step selection function analysis [24] could be applied in future investigations (if available).

Movement distance is predicted to vary with spatial habitat pattern, increasing across disturbed landscapes where risks increase [6]. As the Atlantic Forest and Cerrado biomes continue to fragment, jaguars will likely have to travel longer distances to locate mates and obtain resources. Increased home range size across this lower quality habitat will most likely increase animals’ exposure to risk, including increased vehicle collisions and poaching, and result in a situation often described as an ecological trap [6].

## Conclusions

We compared home range size and movement behavior of jaguars monitored via GPS collars across different temporal periods, unequal sampling intervals, and varying autocorrelation structures. Two factors were critical for this achievement: 1) the joint efforts of researchers working with the species across different sites in Brazil and Argentina, resulting in the largest existing jaguar dataset with over 81,000 locations from 44 tracked individuals; and 2) the use of new analytical methods for movement data. As hypothesized, we observed individual variability on space use and movement, with male jaguars exhibiting larger home ranges, more directional movements, and a higher probability of moving longer daily distances than females. Jaguars inhabiting areas with higher human population size and higher levels of habitat loss were also observed to have larger home ranges Our results fill a gap in the knowledge of the species’ ecology and can contribute to long-term species management and conservation.

## Supporting Information

S1 Table List of the GPS collared jaguars with information on Biome, animal ID, sex and estimated age (years), equipment used (tag brand and satellite system), sampling protocol (time interval between locations), period of data collection, coordinator and institution. (DOCX)

## References

[1] Chapman B, Hulthen K, Wellenreuther M, Hansson L-A, Nilsson JÅ, Bronmark C. Patterns of animal migration In: Hansson L-A, Akesson S, editors. Animal movement across scales. 1st ed Oxford: Oxford University Press; 2014 pp. 11–30.

[2] Nowell K, Jackson P. Wild cats. Status Survey and Conservation Action Plan. IUCN, Gland Switzerland 1996; 110–113.

[3] Macdonald DW. The ecology of carnivore social behaviour. Nature. 1983;301: 379–384.

[4] Crooks KR. Relative sensitivities of mammalian carnivores to habitat fragmentation. Conserv Biol. 2002;16: 488–502.

[5] Cardillo M, Purvis A, Sechrest W, Gittleman JL, Bielby J, Mace GM. Human population density and extinction risk in the world’s carnivores. PLoS Biol. 2004; 10.1371/journal.pbio.0020197 PMC449851 15252445

[6] Fahrig L. Non-optimal animal movement in human-altered landscapes. Funct Ecol. 2007;21: 1003–1015.

[7] Nathan R. An emerging movement ecology paradigm. Proc Natl Acad Sci U S A. 2008;105: 19050–19051. 10.1073/pnas.0808918105 19060197 PMC2614713

[8] Chetkiewicz C-LB, Boyce MS. Use of resource selection functions to identify conservation corridors. J Appl Ecol. 2009;46: 1036–1047.

[9] Rodríguez-Soto C, Monroy-Vilchis O, Maiorano L, Boitani L, Faller JC, Briones MÁ, et al Predicting potential distribution of the jaguar (Panthera onca) in Mexico: Identification of priority areas for conservation. Divers Distrib. 2011;17: 350–361.

[10] Horne JS, Garton EO. Selecting the best home range model: An information-theoretic approach. Ecology. 2006;87: 1146–1152. 16761593 10.1890/0012-9658(2006)87[1146:stbhrm]2.0.co;2

[11] Fleming CH, Fagan WF, Mueller T, Olson KA, Leimgruber P, Calabrese JM. Rigorous home range estimation with movement data: a new autocorrelated kernel density estimator. Ecology. 2015;96: 1182–1188. 26236833 10.1890/14-2010.1

[12] Sanderson EW, Redford KH, Chetkiewicz C-LB, Medellin RA, Rabinowitz AR, Robinson JG, et al Planning to Save a Species: the Jaguar as a Model. Conserv Biol. 2002;16: 1–15. 10.1046/j.1523-1739.2002.00352.x 35701976

[13] Caso A, Lopez-Gonzalez C, Payan E, Eizirik E, de Oliveira T, Leite-Pitman R, et al. Panthera onca. In: The IUCN Red List of Threatened Species [Internet]. 2008 [cited 8 Dec 2015] p. e.T15953A5327466. 10.2305/IUCN.UK.2008.RLTS.T15953A5327466.en.

[14] Morato RG, Beisiegel BDM, Ramalho EE, Boulhosa LRP. Avaliação do risco de extinção da onça-pintada Panthera onca (Linnaeus, 1758) no Brasil. Biodiversidade Bras. 2013;3: 122–132.

[15] G A, E C, De Angelo C, Di Bitetti MS, Lucherini M, Muzzachiodi N, et al Familia: Felidae In: Ojeda R., Chillo V, Isenrath G, editors. Libro Rojo de Mamiferos Amenazados de la Argentina. 1st ed Mendoza: SAREM; 2012 pp. 92–101.

[16] Schaller GB, Crawshaw PGJ. Movement Patterns of Jaguar. Biotropica. 1978;12: 161–168.

[17] Rabinowitz AR, Nottingham BG Jr.. Ecology and behaviour of the Jaguar (Panthera onca) in Belize, Central America. Journal of Zoology. 1986 pp. 149–159.

[18] Astete S, Sollmann R, Silveira L. Comparative Ecology of Jaguars in Brazil. CAT News. 2007; 9–14.

[19] de Azevedo FCC, Murray DL. Spatial organization and food habits of jaguars (Panthera onca) in a floodplain forest. Biol Conserv. 2007;137: 391–402.

[20] Cavalcanti SMC, Gese EM. Spatial ecology and social interactions of jaguars (Panthera onca) in the southern Pantanal, Brazil. J Mammal. 2009;90: 935–945.

[21] Conde DA, Colchero F, Zarza H, Christensen NL, Sexton JO, Manterola C, et al Sex matters: Modeling male and female habitat differences for jaguar conservation. Biol Conserv. 2010;143: 1980–1988.

[22] Colchero F, Conde D a., Manterola C, Chávez C, Rivera a., Ceballos G. Jaguars on the move: Modeling movement to mitigate fragmentation from road expansion in the Mayan Forest. Anim Conserv. 2011;14: 158–166.

[23] Sollmann R, Furtado MM, Gardner B, Hofer H, Jácomo ATA, Tôrres NM, et al Improving density estimates for elusive carnivores: Accounting for sex-specific detection and movements using spatial captureâ€“recapture models for jaguars in central Brazil. Biol Conserv. 2011;144: 1017–1024.

[24] Kanda Z. Ecologia do movimento e dinâmica espaço-temporal da onça pintada no Pantanal sul do Brasil. Universidade Estadual de São Paulo. 2015.

[25] Calabrese JM, Fleming CH, Gurarie E. Ctmm: an R Package for Analyzing Animal Relocation Data As a Continuous-Time Stochastic Process. Methods Ecol Evol. 2016;

[26] Fleming CH, SubaşI Y, Calabrese JM. Maximum-entropy description of animal movement. Phys Rev E—Stat Nonlinear, Soft Matter Phys. 2015;91: 1–6. 10.1103/PhysRevE.91.032107 25871054

[27] Fleming CH, Calabrese JM, Mueller T, Olson K a, Leimgruber P, Fagan WF. From fine-scale foraging to home ranges: a semivariance approach to identifying movement modes across spatiotemporal scales. Am Nat. 2014;183: E154–67. 10.1086/675504 24739204

[28] Fleming CH, Calabrese JM, Mueller T, Olson KA, Leimgruber P, Fagan WF. Non-Markovian maximum likelihood estimation of autocorrelated movement processes. Methods Ecol Evol. 2014;5: 462–472.

[29] Blackwell PG, Niu M, Lambert MS, Lapoint SD. Exact Bayesian inference for animal movement in continuous time. Methods Ecol Evol. British Ecological Society; 2016;7: 184–195.

[30] Crawshaw PG Jr.. Comparative ecology of ocelot (Felis pardalis) and jaguar (Panthera onca) in a protected subtropical forest in Brazil and Argentina. University of Florida 1995.

[31] Cullen L Jr., De Abreu KC, Sana D, Nava AFD. As onças-pintadas como detetives da paisagem no corredor do Alto Paraná, Brasil. Nat e Conserv. 2005;3: 43–58.

[32] de Azevedo FCC, Murray DL. Spatial organization and food habits of jaguars (Panthera onca) in a floodplain forest. Biol Conserv. 2007;137: 391–402.

[33] Silveira L. Ecologia comparada e conservação da onça-pintada (Panthera onca) e onça-parda (Puma concolor), no Cerrado e Pantanal [Internet]. Universidade de Brasília. University of Brasilia. 2004. http://scholar.google.com/scholar?hl=en&btnG=Search&q=intitle:Ecologia+comparada+e+conserva??o+da+on?a-pintada+(Panthera+onca)+e+on?a-parda+(Puma+concolor),+no+Cerrado+e+Pantanal#0

[34] Ribeiro MC, Metzger JP, Martensen AC, Ponzoni FJ, Hirota MM. The Brazilian Atlantic Forest: How much is left, and how is the remaining forest distributed? Implications for conservation. Biol Conserv. Elsevier Ltd; 2009;142: 1141–1153.

[35] Leal I, Silva J, Tabarelli M, Lacre T Jr.. Mudando o curso da conservação da biodiversidade na Caatinga do Nordeste do Brasil. Megadiversidade. 2005;1: 139–146.

[36] Machado RB, Neto MGP, Caldas EF, Gonçalves D a., Santos N a., Tabor K, et al. Estimativas de perda da área do Cerrado brasileiro. … Int do Bras …. 2004; 1–23.

[37] Robinson TP, William Wint GR, Conchedda G, Van Boeckel TP, Ercoli V, Palamara E, et al Mapping the global distribution of livestock. PLoS One. 2014;9. 10.1371/journal.pone.0096084 PMC4038494 24875496

[38] Morato RG, Conforti V a., Azevedo FC, Jacomo a. T a, Silveira L, Sana D, et al Comparative analyses of semen and endocrine characteristics of free-living versus captive jaguars (Panthera onca). Reproduction. 2001;122: 745–751. 11690535 10.1530/rep.0.1220745

[39] Balme GA, Slotow R, Hunter LTB. Impact of conservation interventions on the dynamics and persistence of a persecuted leopard (Panthera pardus) population. Biol Conserv. Elsevier Ltd; 2009;142: 2681–2690.

[40] Sikes R, Gannon W, Mammalogists TAC and UC of the AS of. Guidelines of the American Society of Mammalogist for the use of wild mammals in research. J Mammal. 2011;1: 235–253. 10.1093/jmammal/gyw078 PMC5909806 29692469

[41] Fleming CH, Calabrese JM. ctmm: Continuous-Time Movement Modeling. R package version 0.2.8. [Internet]. CRAN; 2015 http://cran.jellyfish.lol/web/packages/ctmm/index.html

[42] R Development Core Team R. R: A Language and Environment for Statistical Computing [Internet]. Team RDC, editor. R Foundation for Statistical Computing. R Foundation for Statistical Computing; 2011 p. 409.

[43] Akaike H. A new look at the statistical model identification. IEEE Trans Autom Control. 1974;19: 716–723.

[44] Kéry M. Introduction to WinBUGS for Ecologists: A Bayesian approach to regression, ANOVA, mixed models and related analyses. First Edit Academic Press; 2010.

[45] McCarthy MA. Bayesian Methods for Ecology. Cambridge, New York, Melbourne, Madrid, Cape Town, Singapore, Sau Paulo: Cambridge University Press; 2007.

[46] Royston P. The W test for normality. Appl Stat. 1982; 176–180.

[47] Gelman A, Rubin D. Inference from iterative simulation using multiple sequences. Stat Sci. 1992;7: 457–511.

[48] Gelman a, Hill J. Data analysis using regression and multilevel/hierarchical models. Policy Anal. 2007; 1–651.

[49] Plummer M, Best N, Cowles K, Vines K. Coda: Output analysis and diagnostics for MCMC. 2010.

[50] Bright EA, Coleman PR, Rose AN, Urban ML. LandScan 2011. Digit dataset, Oakridge Natl Lab Oakridge, TN, USA, web ornl gov/sci/landscan/index shtml. 2012;

[51] Liu J, Taylor WW, Ouyang Z, Groop R, Tan Y, Zhang H. A framework for evaluating the effects of human factors on wildlife habitat: The case of giant pandas. Conserv Biol. 1999;13: 1360–1370.

[52] De Angelo C, Paviolo A, Wiegand T, Kanagaraj R, Di Bitetti MS. Understanding species persistence for defining conservation actions: A management landscape for jaguars in the Atlantic Forest. Biol Conserv. 2013;159: 422–433.

[53] McCarthy M. Bayesian methods for ecology [Internet]. 1st ed McCarthy MA, editor. Chemistry & biodiversity. New York: Cambridge University Press; 2007.

[54] Crawshaw PG, Quigley HB. Jaguar spacing, activity and habitat use in a seasonally flooded environment in Brazil. J Zool. 1991;223: 357–370. Available: http://doi.wiley.com/10.1111/j.1469-7998.1991.tb04770.x

[55] Powell RA, Mitchell MS. What is a home range? J Mammal. 2012;93: 948–958.

[56] Singh NJNNJ, Börger L, Dettki H, Bunnefeld N, Ericsson G, Borger L, et al From migration to nomadism: Movement variability in a northern ungulate across its latitudinal range. Ecol Appl. 2012;22: 2007–2020. 23210316 10.1890/12-0245.1

[57] Boone RB, Thirgood SJ, Hopcraft JGC. Serengeti wildebeest migratory patterns modeled from rainfall and new vegetation growth. Ecology. 2006;87: 1987–1994. 16937638 10.1890/0012-9658(2006)87[1987:swmpmf]2.0.co;2

[58] Stabach JA, Wittemyer G, Boone RB, Reid RS, Worden J.S.. Variation in habitat selection by white-bearded wildebeest across different degrees of human disturbance. Ecosphere. 2016;

[59] Schaller GB. The Serengeti lion: A study of predator-prey relations [Internet]. University of Chicago, Chicago, Illinois, USA University of Chicago Press; 1976.

[60] Mauritzen M, Derocher AE, Oysten W. Space-use strategies of female polar bears in a dynamic sea ice habitat. Can J Zool. 2001;79: 1704–1713.

[61] May R, van Dijk J, Landa A, Andersen R, Andersen R. Spatio-temporal ranging behaviour and its relevance to foraging strategies in wide-ranging wolverines. Ecol Modell. 2010;221: 936–943.

[62] Kittle AM, Anderson M, Avgar T, Baker JA, Brown GS, Hagens J, et al Wolves adapt territory size, not pack size to local habitat quality. J Anim Ecol. 2015;84: 1177–1186. 10.1111/1365-2656.12366 25757794

[63] Mikael S. The mating tactics and spacing patterns of solitary carnivores In: Gittleman JL, editor. Carnivore behavior, ecology, and evolution. 1st ed New York: Cornell University Press; 1989 pp. 164–182.

[64] De Angelo C, Paviolo A, Di Bitetti M. Differential impact of landscape transformation on pumas (Puma concolor) and jaguars (Panthera onca) in the Upper Paraná Atlantic Forest. Divers Distrib. 2011;17: 422–436.

[65] Carvalho E a. R, Zarco-González MM, Monroy-Vilchis O, Morato RG. Modelling the risk of livestock depredation by jaguar in the Transamazon Highway, Brazil. Basic Appl Ecol. Elsevier GmbH; 2015;16: 413–419.

