# Supplementary material for: Space Use and Movement of a Neotropical Top Predator: The Endangered Jaguar
Source: PLoS One. 2016 Dec 28;11(12):e0168176. doi: 10.1371/journal.pone.0168176 (PMC5193337; doi:10.1371/journal.pone.0168176)
Supplement: S1 Table — (DOCX) [file pone.0168176.s001.docx]

S1. List of the GPS collared jaguars with information on Biome, animal ID, sex and estimated age (years), equipment used (tag brand and satellite system), sampling protocol (time interval between locations), period of data collection, coordinator and institution

| ID | | Sex/age (years) | | Equipment | Sampling protocol | | Start Date | End Date | Coordinator | Institution |  |
| --- | --- | --- | --- | --- | --- | --- | --- | --- | --- | --- | --- |
| *Amazon* | | | | | | | | | | |  |
| *Reserva de Desenvolvimento Sustentável Mamirauá* | | | | | | | | | | |  |
| Baden | | M/9 | | Telonics/Argos | 4h | | 12-03-2014 | 01-15-2015 | Emiliano Esterci Ramalho | Instituto de Desenvolvimento Sustentável Mamirauá |  |
| Caculao | | M/7 | | Telonics/Argos | 4h | | 03-08-2015 | 07-31-2015 |  |  |  |
| Confuso | | M/9 | | Telonics/Argos | 4h | | 11-16-2012 | 09-29-2013 |  |  |  |
| Coto | | F/7 | | Telonics/Argos | 4h | | 02-14-2013 | 07-17-2013 |  |  |  |
| Mamad | | M/7 | | Telonics/Argos | 4h | | 10-21-2013 | 11-08-2014 |  |  |  |
| Mamae | | F/11 | | Telonics/Argos | 4h | | 12-10-2010 | 10-24-2011 |  |  |  |
| Mudinha | | F/5 | | Telonics/Argos | 4h | | 11-12-2012 | 02-25-2014 |  |  |  |
| Perola | | F/5 | | Telonics/Argos | 4h | | 01-17-2015 | 07-25-2015 |  |  |  |
| *Atlantic Forest* | | | | | | | | | | |  |
| *Parque Estadual do Invinhema* | | | | | | | | | | |  |
| Denis | | M/5 | | Followit-GPS | 12h | | 03-22-2005 | 04-10-2006 | Denis Alesio Sana | Instituto Pró-Carnívoros |  |
| Livia | | F/7 | | Followit-GPS | 12h | | 05-21-2004 | 12-16-2004 |  |  |  |
| Taia | | F/4 | | Followit-GPS | 12h | | 02-26-2002 | 04-12-2005 |  |  |  |
| *Parque Estadual do Morro do Diabo* | | | | | | | | | | |  |
| Cassio | | M/6 | | Followit-GPS | 24h | | 01-19-2003 | 06-27-2003 | Laury Cullen Junior/ Fernando Lima | Instituto de Pesquisas Ecológicas |  |
| Femea | | F/5 | | Followit-GPS | 24h | | 07-15-2002 | 11-30-2002 |  |  |  |
| Gigi | | F/7 | | Followit-GPS | 24h | | 09-30-1998 | 07-15-2003 |  |  |  |
| Zezao | | M/8 | | Followit-GPS | 24h | | 05-03-2003 | 21-10-2003 |  |  |  |
| *Parque Nacional Iguazu-Argentina* | | | | | | | | | | |  |
| Guacurari | | M/7 | | Followit-GPS | ½h | | 02-26-2009 | 10-04-2009 | Agustin Paviolo | Instituto de Biologia Subtropical |  |
| Yasirandi | | F/6 | | Lotek-Globalstar | 3h | | 08-24-2012 | 04-06-2013 |  |  |  |
|  | |  | |  |  | |  |  |  |  |  |
| *Parque Nacional do Iguaçu-Brazil* | | | | | | | | | | |  |
| Naipi | | F/2 | | Lotek-Globalstar | 3h | | 02-19-2013 | 06-18-2013 | Marina Xavier da Silva | Parque Nacional do Iguaçu-ICMBio |  |
| Panca | | M/1,5 | | Followit-GPS | 1.5h | | 05-08-2010 | 07-06-2010 |  |  |  |
| Sancho | | M/3,5 | | Followit-GPS | 8h | | 01-27-2012 | 03-13-2012 |  |  |  |
| *Caatinga* | | | | | | | | | | |  |
| Lampiao | | M/10 | | Lotek/Iridium | 1h | | 11-14-2014 | 01-10-2015 | Ronaldo G Morato | Centro Nacional de Pesquisa e Conservação de Mamíferos Carnívoros (CENAP)-ICMBio |  |
| Courisco | | M/5 | | Lotek/Iridium | 1h | | 09-16-2015 | 02-06-2016 |  |  |  |
| *Cerrado* | | | | | | | | | | |  |
| Xango 1 | | M | | Northstar/  Globalstar | 1h | | 06-06-2013 | 02-06-2014 | Cristina Gianni/ Marina Motta Carvalho | NEX |  |
| Xango 2 | | M | | Northstar/  Globalstar | 3h | | 04-24-2015 | 10-20-2015 |  |  |  |
| *Pantanal* | | | | | | | | | | |  |
| Alice | | F/4 | | Lotek/Iridium | 1h | | 12-05-2014 | 04-18-2015 | Daniel Kantek/ Selma Onuma/ Ronaldo G Morato | Estação Ecológica Taiamã/CENAP-ICMBio |  |
| Anderson | | M/7 | | Lotek/Iridium | 1h | | 12-07-2014 | 08-24-2015 |  |  |  |
| Caiman | | M/5 | | Lotek/Iridium | 1h | | 11-29-2014 | 04-13-2015 |  |  |  |
| Dale | | M/7 | | Lotek/Iridium | 1h | | 09-11-2014 | 05-21-2015 |  |  |  |
| Daryl | | M/5 | | Lotek/Iridium | 1h | | 09-01-2014 | 09-26-2014 |  |  |  |
| Fera | | F/3 | | Lotek/Iridium | 1h | | 12-05-2014 | 08-17-2015 |  |  |  |
| Linda | | M/5 | | Lotek/Iridium | 1h | | 11-28-2014 | 12-25-2014 |  |  |  |
| Milagre | | M/6 | | Lotek/Iridium | 1h | | 10-11-2015 | 12-10-2015 |  |  |  |
| Picole | M/4 | | Lotek/Iridium | | | 2h | 10-15-2013 | 05-29-2015 |  | | |
| Selema | F/6 | | Lotek/Iridium | | | 1h | 11-10-2015 | 12-11-2015 |  |  |  |
| Wendy | F/5 | | Lotek/Globalstar | | | 2h | 10-09-2013 | 04-19-2014 |  |  |  |

Continue

| *Pantanal* | | | | | | | |
| --- | --- | --- | --- | --- | --- | --- | --- |
| Brazuca | M/5 | Lotek/Iridium | 1h | 04-04-2012 | 04-15-2012 | Mario Haberfeld/ Rogerio Cunha de Paula | Projeto Onçafari/CENAP-ICMBio |
| Brutus | M/5 | Lotek/Iridium | 1h | 10-19-2013 | 01-03-2014 |  |  |
| Chuva | F/10 | Lotek/Iridium | 1h | 10-30-2011 | 01-11-2012 |  |  |
| Esperanca 1 | F/7 | Lotek/Iridium | 1h | 10-22-2012 | 12-14-2012 |  |  |
| Esperanca 2 | F/10 | Lotek/Iridium | 1h | 04-26-2015 | 08-30-2015 |  |  |
| Nati | M/10 | Lotek/Iridium | 1h | 11-01-2011 | 12-23-2011 |  |  |
| Natureza | F/2 | Lotek/Iridium | 1h | 10-27-2013 | 05-13-2014 |  |  |
| Nusa | F/10 | Lotek/Iridium | 1h | 04-20-2015 | 08-25-2015 |  |  |
| Teorema | F/7 | Lotek/Iridium | 1h | 04-21-2013 | 01-21-2014 |  |  |
| Troncha | F/10 | Lotek/Iridium | 1h | 10-22-2013 | 01-17-2014 |  |  |
| Vida | F/5 | Lotek/Iridium | 1h | 05-15-2012 | 06-17-2012 |  |  |
